# Feeding practices of under-two children in Ethiopia: A systematic review and meta-analysis

**DOI:** 10.1371/journal.pone.0342932

**Published:** 2026-02-25

**Authors:** Sisay Eshete Tadesse, Amare Tariku, Tefera Belachew

**Affiliations:** 1 School of Public Health, College of Medicine and Health Sciences, Wollo University, Dessie, Ethiopia; 2 Department of Nutrition and Dietetics, Faculty of Public Health, Institute of Health, Jimma University, Jimma, Ethiopia; 3 Department of Nutrition and Dietetics, Institute of Public Health, College of Medicine and Health Sciences, University of Gondar, Gondar, Ethiopia; Mizan-Tepi University, ETHIOPIA

## Abstract

**Background:**

Globally, child feeding practices remain suboptimal and contribute substantially to a high burden of malnutrition. In Ethiopia, evidence on the overall status of feeding practices among children under two years of age is limited. This study therefore sought to estimate the pooled prevalence of timely initiation of breastfeeding, exclusive breastfeeding, timely initiation of complementary feeding, and minimum acceptable diet, as well as to identify the factors associated with these practices.

**Method:**

This systematic review and meta-analysis was conducted following the Preferred Reporting Items for Systematic Review and Meta-Analysis guidelines. A comprehensive literature search was performed in Scopus, HINARI, the Cochrane Library, and PubMed/MEDLINE. Cross-sectional and case-control studies published in English were included. Study quality was assessed using the Joanna Briggs Institute critical appraisal checklist. Heterogeneity across studies was assessed using Cochran Q test and the I^2^ statistic. Data analysis was done using STATA/MP version 17.0. Adjusted odds ratios with 95% confidence intervals were used to identify factors. Publication bias was assessed using funnel plots, Egger weighted regression, and Begg rank correlation tests.

**Results:**

A total of 107 articles were included in this study. The pooled prevalence of timely initiation of breastfeeding, exclusive breastfeeding, timely initiation of complementary feeding, and optimal feeding practice were 64% (95% CI: 50%, 78%), 58.6% (95% CI: 52.6%, 64.5%), 60.51% (95% CI: 54.2%, 66.9%), and 20.4% (95% CI: 13.6%, 27.0%), respectively. Antenatal care (AOR = 3.4; 95% CI: 1.5, 7.5), place of delivery (AOR = 2.3; 95% CI: 1.1, 4.9), and normal delivery (AOR = 3.3; 95% CI: 1.1, 10) were positively associated with timely initiation of breastfeeding. Exclusive breastfeeding was positively associated with infant age 0–1 (AOR = 4.4; 95% CI: 1.4, 13.6) and 2–3 months (AOR = 2.5; 95% CI: 1.2, 5.1), maternal age > 35 (AOR = 3.4; 95% CI: 1.3, 8.7), residence (AOR = 1.8; 95% CI: 1.1, 3.1), maternal occupation (AOR = 1.8; 95% CI: 1.2, 2.7), place of delivery (AOR = 2.1; 95% CI:1.2, 3.7), normal delivery (AOR = 1.7; 95 CI:1.2, 2.6), postnatal care (AOR = 2.3; 95% CI: 1.2, 4.3), counseling (AOR = 2.3; 95% CI:1.4, 3.9) and husband support (AOR = 2.9; 95% CI:1.9, 4.4) were positively associated with EBF. Antenatal care (AOR = 3.4; 95% CI:1.5, 7.5) and place of delivery (AOR = 2.3; 95% CI:1.1, 4.9) were positively associated with timely initiation of complementary feeding. Optimal infant feeding practice was positively associated with nutrition education through demonstrations (AOR = 2.1; 95% CI:1.3, 3.3) and age of child 18–23 months (AOR = 2.7; 95% CI:1.2, 6.1).

**Conclusion:**

This study demonstrated that infant feeding practices were below both national and international recommendations, exposing children at higher risk of malnutrition, morbidity, and mortality. These suboptimal feeding practices also hinder progress toward achieving to achieving the Sustainable Development Goals. Several factors were identified as significant determinants of feeding practices, including antenatal care attendance, place and mode of delivery, postnatal care utilization, maternal occupation, maternal age, child’s age, breastfeeding counseling, husband’s support, place of residence, and participation in complementary food preparation demonstrations. Improving infant feeding practices therefore requires a comprehensive approach. Key strategies to improve infant feeding practices should include nutrition education through practical demonstrations, promoting full attendance of antenatal and postnatal care, increasing institutional deliveries, enhancing husband involvement, and tailoring interventions to the child’s age as well as the mother’s age and place of residence.

## Introduction

Infant feeding practices are key determinants of child health and development. The World Health Organization (WHO) recommends exclusive breastfeeding for the first six months of life, followed by the introduction of complementary foods while continuing breastfeeding up to two years of age or beyond [1]. Globally, nearly half (49%) and 44% of the newborns-initiate breastfeeding within one hour of birth and exclusively breastfed for the first six months, respectively [2]. According to the 2020 global nutrition report, only 18.9% of children aged 6–23 months receive a nutritionally adequate diet [3]. In developing countries, 42% of infants are exclusively breastfed for six months of age, and 73% of children aged 6–8-months receive solid, semisolid, and soft foods [2].

Sub-optimal infant feeding practices are strongly linked to high morbidity and mortality among children. Most growth deficits occur between 6–12 months of age [4] and inappropriate feeding practices contribute up to a third of all cases of child malnutrition [5]. Current data suggest that over 1 million children die each year due to wasting [6]. Many children under-two-year experience delayed introduction of complementary foods, are fed infrequently, or receive foods of inadequate quality [7–9]. Such sub-optimal feeding practices contribute to feeding difficulties, increased risk of infection and allergies, early cessation of breastfeeding, undernutrition, obesity in childhood and adulthood, reduced breast milk intake, and long-term impacts on feeding performance [10,11].

Previous studies have shown that maternal education and age, antenatal care, postnatal care, place of delivery, household wealth, place of residence, maternal employment, household size, and father’s education all significantly influence feeding behaviors [12–15].

Despite the well-established benefits of optimal infant feeding practices, suboptimal practices remain widespread in many regions, including Ethiopia. The 2019 Ethiopian Demographic and Health Survey (EDHS) reported that only 59% of infants under six months were exclusively breastfed, and merely 11.3% of children aged 6–23 months received a minimum acceptable diet [16]. World Bank estimates indicate that undernourished children may lose over 10% of their potential lifetime earnings, adversely affecting national productivity [17]. Although many studies were conducted on infant feeding practices, there were inconsistencies across studies, posing challenges for policymakers, practitioners, and stakeholders in making evidence-based decisions.

Previous systematic reviews and meta-analyses on child feeding practices in Ethiopia have several limitations. For instance, a systematic review and meta-analysis (SRMA) on timely initiation of breastfeeding (TIBF) included a retracted article and focused specifically on the effects of cesarean delivery and kangaroo mother care on preterm and low-birth-weight infants [13]. Similarly, a SRMA on exclusive breastfeeding (EBF) examined only a single influencing factor [14,15]. A SRMA on a minimum acceptable diet (MAD) included only nine studies and didn’t address the determinants [18]. Moreover, earlier SRMA predominantly included cross-sectional studies, limiting the comprehensiveness of the evidence.

The current study addresses the gaps identified in previous SRMA by incorporating a larger number of studies (107) including both case-control and cross-sectional designs, and examining the determinants of infant feeding practices. It also considers four core IYCF indicators: TIBF, EBF, TICF and MAD. This SRMA provides a comprehensive and rigorous synthesis of the available evidence on child feeding practices and will inform decision-making, guide resource allocation, and offer new insight for designing interventions to improve IYCF practices and reduce the burden of malnutrition in Ethiopia.

## Materials and methods

### Study setting

This systematic review and meta-analysis was conducted in Ethiopia, a country located in the northeastern part of Africa, also known as the Horn of Africa, and one of the most populous nations in the region. Traditional child feeding malpractices such as prelacteal feeding, discarding colostrum and inappropriate timing of complementary feeding remain a major concern, adversely affecting the health of many newborns and young children. Since 2000, efforts to reduce child malnutrition have been implemented through various intervention strategies, including the IYCF program, Seqota declaration, scaling up nutrition, multisectoral nutrition coordination, food and nutrition policy, and the National Nutrition Program, in collaboration with international partners. Similarly, one of the health packages that the country is working on is minimizing inappropriate child feeding and other maladaptive practices.

### Search strategy

This systematic review and meta-analysis was performed according to the Preferred Reporting Items for Systematic Review and Meta-Analysis (PRISMA) guidelines [19]. Three reviewers performed a comprehensive search of relevant articles across multiple databases, including MEDLINE (via PubMed), EMBASE, Cochrane Library, SCOPUS, HINARI, and Google Scholar. In addition, gray literature was identified through manual searches.

### Searching terms

Search strategies for each database were developed based on the CoCoPop framework:

**Condition:** studies documenting infant feeding practices

**Context:** studies conducted in Ethiopia

**Population:** Studies targeting children under two years of age were included

Relevant articles were searched using the following searching terms: (prevalence) OR (magnitude) AND (determinants) OR (“associated factors”) OR (“risk factors”) AND (“infant feeding practices”) AND (“exclusive breastfeeding”) OR (complementary feeding*) OR (“minimum acceptable diet”) OR (optimum nutrition) AND (Ethiopia). Since no eligible studies were found prior to 2011, only articles published from 2011 onwards were included. This SRMA was registered in PROSPERO with a CRD number of 42023489496. No separate study protocol was prepared.

### Eligibility criteria

All studies conducted in Ethiopia that reported prevalence, determinants and factors associated with infant feeding practices, which were published in English were included. Both cross-sectional and case-control study designs were considered. Studies were excluded if the full text was unavailable after attempting to contact the primary investigator for three months, systematic reviews of interventions, review articles, conference abstracts, and editorials.

### Data extraction

After obtaining the full texts, duplicates were identified and removed using EndNote. Data extraction was conducted independently by three reviewers (SE, TB, and AT), who screened titles, abstracts, and full articles. The study selection procedure is presented using a PRISMA flow diagram [19]. Studies that met the inclusion criteria were retained, while those “included” and “undecided” studies underwent further full-text assessment. To minimize bias, reviewers independently ranked articles without knowing each other’s decisions, and any discrepancies were resolved through discussion and consensus. Data were then extracted using a Microsoft Excel 2021 extraction sheet, capturing study characteristics (author, year of publication, region, target population, sample size, study design, and response rate), infant feeding practices, subject recruitment procedures, adjusted odds ratios, and population characteristics.

### Quality assessment and risk of bias

The quality of included studies was assessed independently by three investigators using the Joanna Briggs Institute (JBI) critical appraisal checklist, and the quality scores were averaged. Any disagreement was resolved through discussion and consensus. During data extraction data quality was ensured by selecting reliable and relevant data sources, removing duplicates and cross-checking extracted data against the original article. Finally, studies with a score of 50% and above were included in this systematic review and meta-analysis.

### Data synthesis and analysis

Data were analyzed using STATA/MP version 17.0. The prevalence of infant feeding practices was reported by the forest plots. To estimate the determinants of feeding practices, adjusted odds ratio with a 95% confidence interval (CI) was pooled. Heterogeneity between studies was assessed using the Cochrane Q statistic (significant if the P-value was < 0.05) and quantified with the I² statistic (at least 50%, was considered suggestive of statistically significant heterogeneity) [20].

A Der Simonian and Laird random-effects model was used for studies with high heterogeneity, and a fixed effects model with inverse variance methods was used for similar studies. Heterogeneity was further explored using the Galbraith plot and Forest plot. The source of heterogeneity was tested by running meta-regression, subgroup analysis by sex, age and region), and sensitivity analysis. Publication bias was assessed by funnel plots, Egger weighted regression, and Begg rank correlation tests at p-values < 0.05.

## Results

### Study characteristics

**Fig 1:**
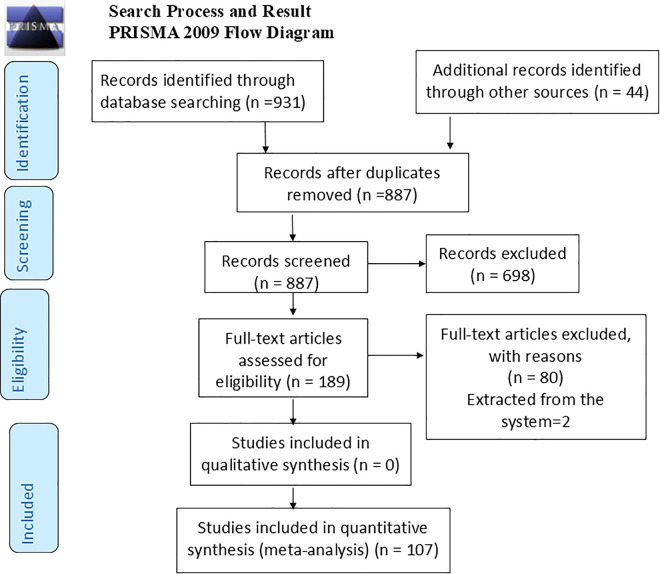
Preferred Reporting Items for Systematic Review and Meta-Analysis for literature screening and selection process, June 2020.

A total of 975 articles were retrieved from databases by a literature search (Fig 1).

After screening the title and abstract, 189 articles were identified as having information relevant to infant feeding practices. Following full text review, 107 articles were evaluated to have better quality data that met the criteria for abstraction. Of these, 18 studies involving 16,405 participants were included for TIBF [21–33]; 39 papers with 19,987 participants addressed EBF [34–63]; 26 papers involving 15,855 participants assessed TIBF [64–79]; and 24 papers including 15, 495 infants aged 6–23 months were included for assessing MAD [64,75, 80–99]. All included articles were full-text articles employing cross-sectional and case-control designs and were published between 2011 and 2024 (Table 1).

**Table 1:** Summary of Extracted Studies on Infant Feeding Practices among Under-two Children in Ethiopia.

Author	Year	Setting	Study Design	Region	Sample Size	Prev	Target	Quality
							Group	
Tewabe, T., et al	2016	Community-based	Cross-sectional	Amhara	423	79	< 6 months	56
Ayalew, T., et al	2019	Community-based	Cross-sectional	Amhara	423	65	< 6 months	66
Gebremeskel, S. G., et al	2019	Community-based	Cross-sectional	Tigray	803	61	< 12 months	58
Gebretsadik, G. G., et al	2020	Community-based	cross sectional	Afar	390	64	< 12 months	75
Mengistu, G. T., et al	2023	Community-based	cross sectional	National	1420	89	< 24 months	70
Liben, M. L., et al	2016	Community-based	cross sectional	Afar	407	94	< 24 months	80
Gebretsadik, G. G., et al	2023	Community-based	cross sectional	Tigray	633	58	< 6 months	67
Tariku, A., et al	2017	Community-based	cross sectional	Amhara	822	53	6-59 months	73
Setegn T., et al	2011	Community-based	Mixed CS	Oromia	608	52	< 12 months	78
Hailemariam W.T., et al	2015	Community-based	cross sectional	Oromia	593	83	< 24 months	56
Berhe H., et al	2012	Community-based	cross sectional	Tigray	361	78	< 12 months	50
Alemayehu M., et al	2014	Community-based	cross sectional	Tigray	418	42	< 12 months	63
Adugna T.D., et al	2014	Community-based	cross sectional	SNNP	383	43	< 12 months	65
Gargamo BD., et al	2020	Community-based	cross sectional	SNNP	383	80	< 12 months	60
Shiferaw R., et al	2021	Institution-based	cross sectional	Amhara	421	57	New born infants	50
Gedefaw G., et al	2020	Community-based	cross sectional	National	7115	25	6-59 months	75
Tilahun G., et al	2016	Community-based	cross sectional	Amhara	416	63	< 6 months	50
Woldemichael B., et al	2016	Community-based	cross sectional	Oromia	386	67	< 12 months	80
Seid A. M., et al	2013	Community-based	cross-sectional	Amhara	819	50	< 12 months	80
Belachew A., et al	2018	Community-based	cross-sectional	Amhara	499	86	< 6 months	90
Sonko A., et al	2015	Community-based	Cross-sectional	SNNP	422	71	< 6 months	65
Hagos D., et al	2020	Community-based	Cross-sectional	SNNP &Tigray	572	88	< 6 months	75
Teka, B., et al	2015	Community-based	Cross-sectional	SNNP	541	70	< 6 months	80
Egata, G., et al	2013	Community-based	Cross-sectional	Tigray	541	28	< 24 months	63
Tadesse, T., et al	2016	Community-based	Cross-sectional	SNNP	602	49	< 6 months	73
Feleke, D. G., et al	2021	Community-based	Cross-sectional	Amhara	860	40	6–12 months	54
Mekebo, G. G., et al	2022	Community-based	Cross-sectional	National	566	83	< 6 months	65
Asfaw, M. M., et al	2015	Community-based	Cross-sectional	Amhara	634	69	< 12 months	68
Setegn, T., et al	2012	Community-based	Cross-sectional	Oromia	608	71	< 12 months	69
Liben, M. L., et al	2016	Community-based	Cross-sectional	Afar	333	81	< 6 months	68
Shifraw, T., et al	2015	Institution-based	Cross-sectional	AA	648	29	< 9 months	74
Gizaw, Z., et al	2017	Community-based	Cross-sectional	Afar	254	74	6-23 months	75
Yimer, D. S., et al	2021	Community-based	Cross-sectional	Amhara	700	46	6-23 months	65
Shitie, A., et al	2022	Community-based	Cross-sectional	Somali	532	52	6-12 months	74
Tewabe, T., et al	2016	Community-based	Cross-sectional	Amhara	423	50	< 6 months	81
Mebratu, L., et al	2020	Institution-based	Cross-sectional	SNNP	209	82	<18 months (HIV exposed)	65
Jama, A., et al	2020	Community-based	Cross-sectional	Somali	474	20	6-24 months	64
Gebrekidan, K., et al	2021	Community-based	Cross-sectional	Tigray	510	56	6-24 months	59
Kebede, T., et al	2020	Community-based	Cross-sectional	Oromia	313	76	6-24 months	56
Chekol, D. A., et al	2017	Community-based	CCS	Amhara	649	41	7-12 months	60
Tadesse, F., et al	2019	Community-based	CCS	Somali	558	71	3-5 months	53.5
Hunegnaw, M. T., et al	2017	Community-based	Cross-sectional	Amhara	506	74	6-12 months	71
Mekuria, G., et al	2015	Community-based	Cross-sectional	Amhara	423	61	< 6 months	66
Zewdie, A., et al	2022	Community-based	CCS	SNNP	485	64	6-12 months	72
Asemahagn, M. A., et al	2016	Community-based	Cross-sectional	Amhara	346	79	< 6 months	61
Lenja, A., et al	2016	Community-based	Cross-sectional	SNNP	396	78	< 6 months	80
Awoke, S., et al	2021	Community-based	Cross-sectional	SNNP	630	46	< 6 months	80
Getachew, D., et al	2023	Institution-based	Cross-sectional	Somali	336	106	< 6 months	72
Tiruye, G., et al	2018	Institution-based	Cross-sectional	Oromia	422	43	< 15 months	68
Jebena, D. D., et al	2022	Community-based	Cross-sectional	Oromia	649	70	< 6 months	72
Dachew, B. A., et al	2014	Institution-based	Cross-sectional	Amhara	178	36	< 6 months	75
Mamo, K., et al	2020	Community-based	Cross-sectional	Oromia	710	65	6-9 months	60
Bewket Zeleke, L., et al	2017	Community-based	Cross-sectional	Amhara	351	24	6-23 months	50
Yimer D. S., et al	2021	Community-based	Cross-sectional	Amhara	700	46	6-24 months	63
Elyas L., et al	2019	Facility-based	Cross-sectional	AA	380	44.2	6-9 months	75
Kalaye T., et al	2017	Community-based	cross-sectional	SNNP	421	64.8	6-23 months	60
Shiferaw ZB., et al	2017	Community-based	cross-sectional	SNNP	765	49.2	6-23 months	65
Yohannes, B., et al	2018	Community-based	Cross-sectional	SNNP	543	34	6–24 months	70
Abate, A. D., et al	2023	Community-based	Cross-sectional	Amhara	770	71	6–24 months	56
Andualem, A., et al	2020	Institution-based	Cross-sectional	Amhara	280	65	6–24 months	72
Shumey, A., et al	2013	Institution-based	Cross-sectional	Tigray	422	63	6–24 months	68
Mohammed, S., et al	2018	Institution-based	Cross-sectional	AA	600	83	6–24 months	75
Biks, G. A., et al	2018	Community-based	Cross-sectional	Amhara	591	54	6–24 months	72
Sisay, W., et al	2016	Community-based	Cross-sectional	Amhara	421	63	6–24 months	65
Gilano, G., et al	2022	Community-based	Cross-sectional	National	4061	36	6–24 months	65
Tadesse, M., et al	2023	Community-based	Case control	Amhara	482	241	6–24 months	50
Ayana, D., et al	2017	Community-based	Cross-sectional	B. Gumuz	806	62	6–24 months	63
Semahegn, A., et al	2014	Institution-based	Cross-sectional	Harari	200	61	6–24 months	50
Hailu, D., et al	2021	Community-based	Cross-sectional	Amhara	414	49	6–24 months	56
Yeshaneh, A., et al	2021	Community-based	Cross-sectional	Amhara	264	27	6–24 months	60
Kassa, T., et al	2016	Community-based	Cross-sectional	Oromia	611	73	6–24 months	63
Ahmed J., et al	2022	Community-based	Cross-sectional	Oromia	536	68	6–24 months	68
Yeheyis T., et al	2015	Institution-based	Cross-sectional	AA	400	55	6-12 Months	70
Agedew E., et al	2014	Community-based	Cross-sectional	SNNP	562	60	6-12 Months	75
Hibstu T.D., et al	2018	Community-based	Cross-sectional	SNNP	320	58	6–24 months	65
Yazew G.K., et al	2019	Institution-based	Cross-sectional	Amhara	381	66	6–24 months	60
Gessesse D., et al	2013	Community-based	Cross-sectional	Amhara	554	56	6–24 months	63
Neme K., et al	2017	Community-based	Cross-sectional	Oromia	260	40	6–24 months	68
Mekbib E., et al	2014	Community-based	Cross-sectional	Tigray	434	80	6–24 months	65
Chane T., et al	2017	Community-based	Cross-sectional	SNNP	623	71	6–24 months	72
Yemane S., et al	2014	Community-based	Cross-sectional	Tigray	422	53	6–24 months	63
Tafesse T., et al	2018	Community-based	Cross-sectional	SNNP	503	57	6–12 months	67
Akalu E., et al	2017	Institution-based	Cross-sectional	AA	395	67	6–12 months	60
Mulat, E., et al	2019	Community-based	Cross-sectional	Amhara	506	9	6-23 months	55
Ahmed, K. Y., et al	2020	Community-based	Cross-sectional	national	2864	7	7-23 months	50
Teshome, F., et al	2022	Community-based	Cross-sectional	national	1457	11	6-23 months	55
Molla, A., et al	2021	Community-based	Cross-sectional	Amhara	531	32	6-23 months	50
Yisak, H., et al	2020	Institution-based	Cross-sectional	Amhara	287	35	6–24 Months (HIV-Exposed)	58
Abebe, H., et al	2021	Institution-based	Cross-sectional	AA	575	75	6-23 months	75
Birhanu, H., et al	2022	Community-based	Cross-sectional	Amhara	738	19	6-23 months	70
Birie, B., et al	2021	Community-based	Cross-sectional	Amhara	430	13	6-23 months	80
Dejene Y., et al	2023	Community-based	Cross-sectional	Amhara	387	17	6-23 months	67
Farah, S., et al	2024	Community-based	Cross-sectional	Somali	536	47	6-23 months	73
Belete, S., et al	2022	Community-based	CCS	Amhara	732	18	6-23 months	78
Gebretsadik, M. T., et al	2023	Community-based	Cross-sectional	Oromia	845	9	6-23 months	60
Birhanu M.	2017	Community-based	Cross-sectional	Sidama	675	9	6-23 months	56
Kassa, T.	2016	Community-based	Cross-sectional	Oromia	611	10	6–23 months	50
Fanta, M.	2020	Community-based	Cross-sectional	Oromia	325	10	6–23 months	63
Areja, A., et al	2017	Community-based	Cross-sectional	SNNP	546	11	6–23 months	65
Epheson, B., et al	2018	Community-based	Cross-sectional	SNNP	401	9	6–23 months	60
Dagne, A. H.	2019	institution-based	Cross-sectional	Amhara	409	37	6–23 months	50
Demilew, Y. M.	2017	Community-based	Cross-sectional	Amhara	423	7	6–24 months	50
Isse A.A., et al	2023	Community-based	Cross-sectional	Somali	292	22	6–24 months	56
Gizaw G., et al	2019	Institution-based	Cross-sectional	Oromia	200	13	6–24 months	60
Feleke WF., et al	2020	Community-based	Cross-sectional	SNNP	662	36	6–24 months	63
Ergib M., et al	2014	Community-based	Cross-sectional	Tigray	434	11	6–24 months	68
Molla K., et al	2019	Community-based	Cross-sectional	Afar	632	9	6–24 months	70

### Pooled prevalence of infant feeding practices

**Fig 2:**
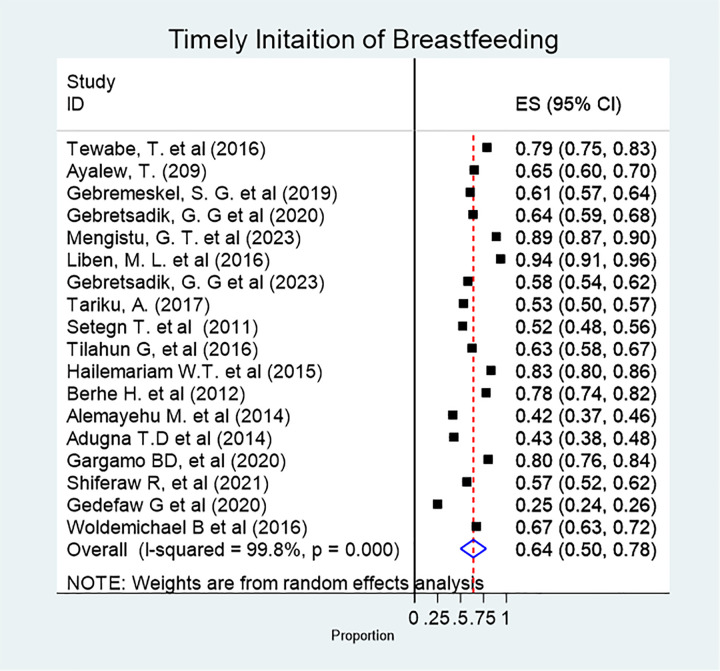
Forest plot showing the pooled prevalence of timely initiation of breastfeeding among infants in Ethiopia, 2024.

The pooled prevalence of TIBF was 64% (95% CI: 50, 78%). There was substantial heterogeneity among the included studies (χ² = 1404.11; p < 0.0001; I^2^ = 99.7%) (Fig 2).

Based on the subgroup analysis, the highest (77.8%) and lowest (47.4%) prevalence of TIBF was reported from Afar and Somali regions, respectively.

**Fig 3:**
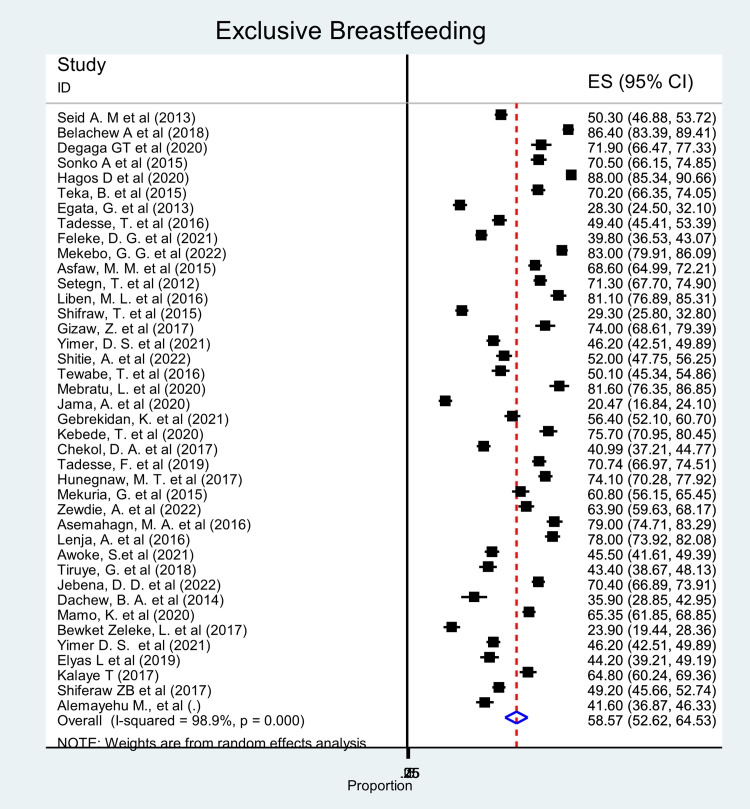
Forest plot showing the pooled prevalence of exclusive breastfeeding among children in Ethiopia, 2024.

The pooled prevalence of EBF among under-two children was 59.6% (95% CI: 52.6, 64.5%). Significant heterogeneity was observed across studies (χ² = 3561.76; p < 0.0001; I^2^ = 98.9%, p = 0.0001) (Fig 3).

Based on the subgroup analysis, the highest (79%) and lowest (23.9%) prevalence was reported from the Amhara region. The subgroup analysis was done by region and study setting, and still, there is high heterogeneity.

**Fig 4:**
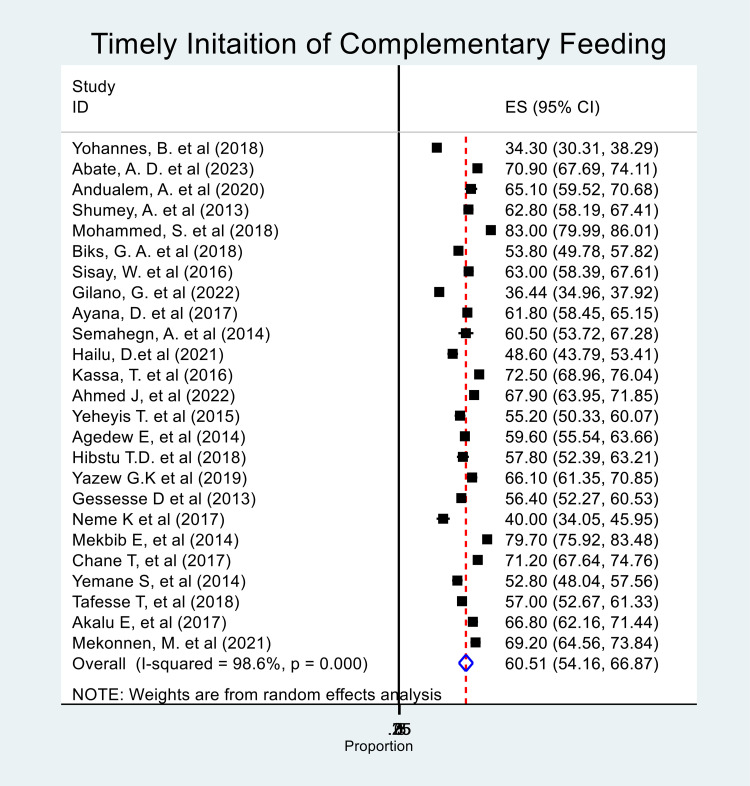
Forest plot showing the pooled prevalence of timely initiation of complementary feeding among infants in Ethiopia, 2024.

The pooled prevalence of TICF was 60.5% (95% CI: 54.2, 66.9%) with high heterogeneity between studies (chi-squared = 6066.37, I-squared = 99.6%%, p < 0.001) (Fig 4).

**Fig 5:**
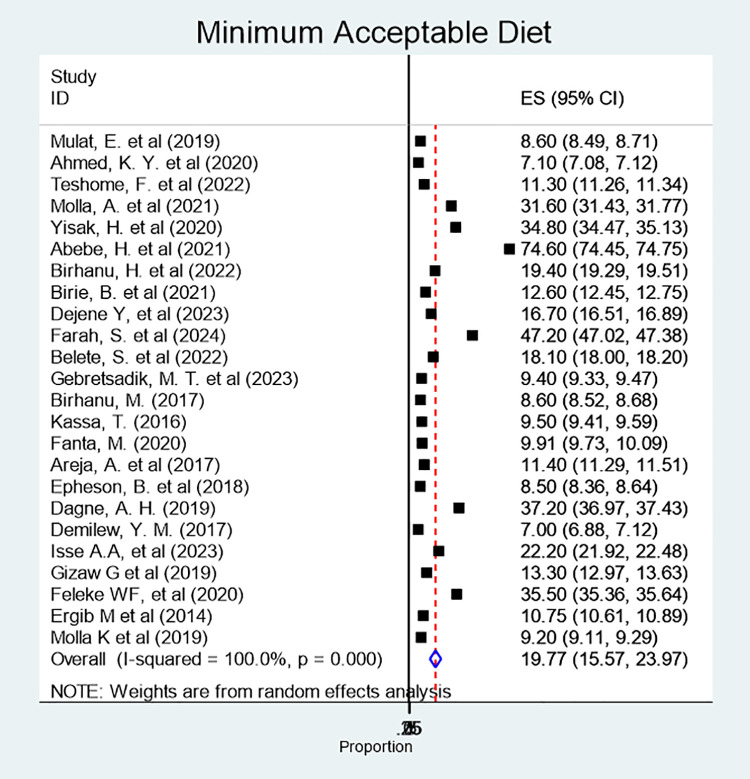
Forest plot showing the pooled prevalence of minimum acceptable diet among children aged 6-23 months, 2024.

The pooled prevalence of MAD among children aged 6–23 months was 19.8% (95% CI: 15.6, 23. 9%) (Fig 5).

The subgroup analysis demonstrated that studies conducted in the Amhara region and those conducted at the national level were major contributors for the observed heterogeneity.

### Sensitivity analysis

The sensitivity analysis demonstrated that the quality score didn’t affect the outcome of the meta-analysis and there was no significant difference in the overall pooled prevalence.

### Publication bias

**Fig 6:**
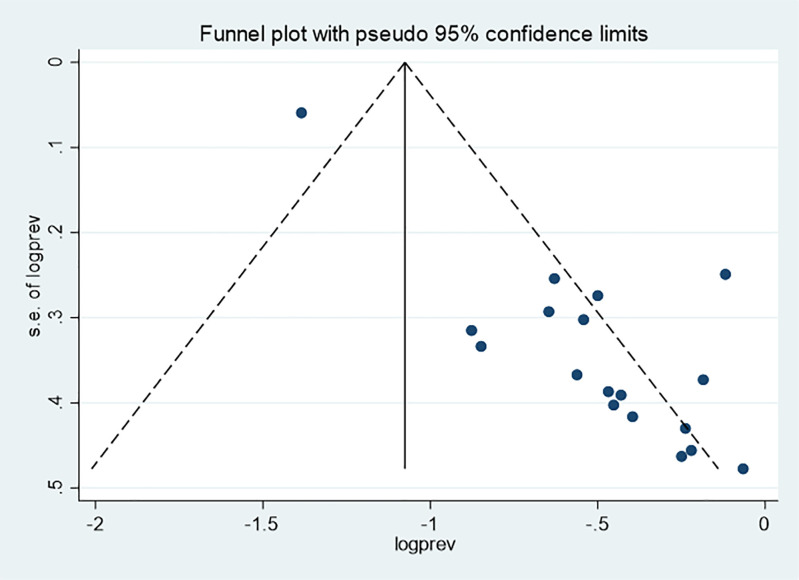
Funnel plot for timely initiation of breastfeeding among infants in Ethiopia, 2024.

Publication bias was assessed both subjectively using a funnel plot and objectively using Begg’s and Egger’s tests. For TIBF, the funnel plot and Egger’s test (p = 0.011) suggested the presence of publication bias, whereas Begg’s test indicated no evidence of publication bias (p = 0.289) (Fig 6).

**Fig 7:**
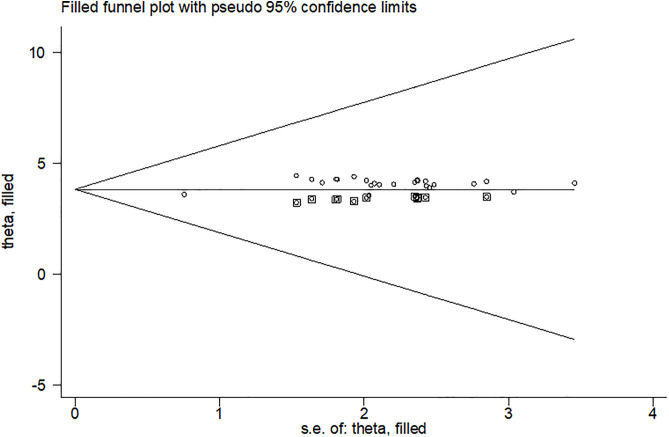
Funnel plots based on trim and fill analysis for the timely initiation of breastfeeding in children aged 6-23 months in Ethiopia, 2024.

Consequently, a trim and fill analysis was done for TIBF (Fig 7).

**Fig 8:**
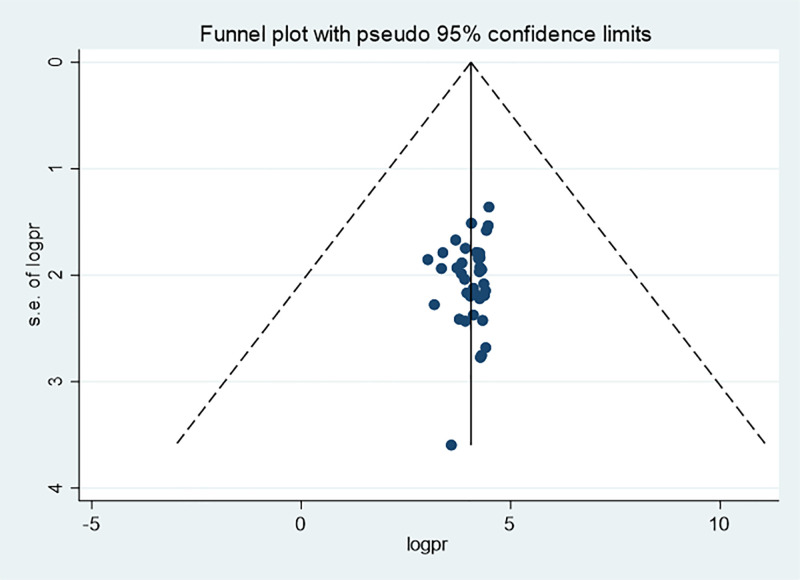
Funnel plot for exclusive breastfeeding of infants in Ethiopia, 2024.

The funnel plot for EBF visually suggested the presence of publication bias, however, both Begg’s (p = 0.556) and Egger’s (p = 0.433) tests didn’t indicate statistically significant publication bias (Fig 8).

**Fig 9:**
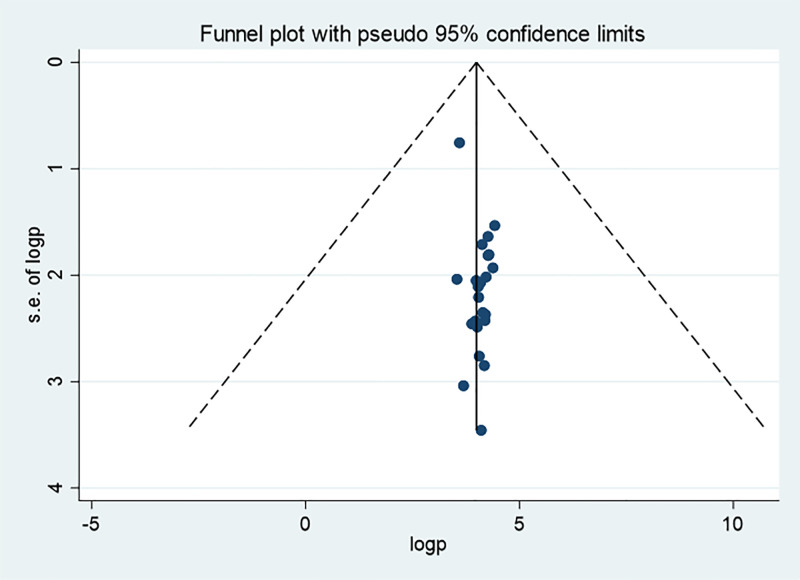
Funnel Plot for the timely initiation of complementary feeding among children aged 6-23 months in Ethiopia, 2024.

Regarding TICF, neither the funnel plot nor Begg’s (p = 0.056) and Egger’s (p = 0.060) tests showed evidence of publication bias (Fig 9).

**Fig 10:**
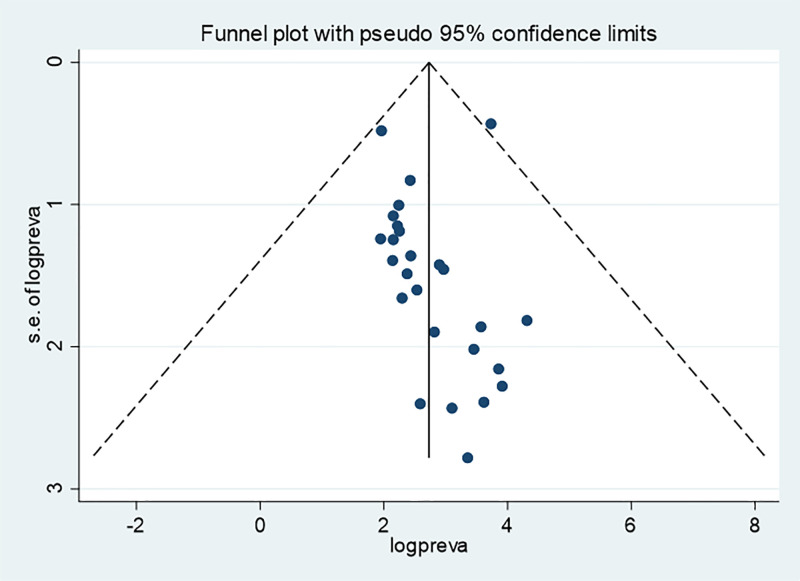
Funnel plot for minimum acceptable diet among children aged 6–23 months in Ethiopia, 2024).

Similarly, the funnel plot for MAD practices showed the presence of possible publication bias; but Begg’s (p = 0.89) and Egger’s (p = 0.934) tests revealed no statistically significant publication bias (Fig 10).

### Factors associated with feeding practices of under-two children

Mothers who had antenatal care follow-up were almost three times more likely to initiate breastfeeding early compared with those without antenatal care (AOR = 3.4%; 95% CI: 1.5, 7.5). Mothers who delivered by cesarean section were 70% less likely to initiate breastfeeding timely than those who had vaginal delivery (AOR = 0.3; 95% CI: 0.1, 0.9). Similarly, facility-based delivery was associated with higher odds of timely initiation of breastfeeding compared with home delivery (AOR = 2.3; 95% CI: 1.1, 4.9) (Table 2).

**Table 2:** Determinants of feeding practices among under-two children in Ethiopia, 2024.

Variables		Pooled AOR (95% CI) for TIBF	Heterogeneity		Pooled AOR (95% CI) for EBF	Heterogeneity		Pooled AOR (95% CI) for TICF	Heterogeneity		Pooled AOR (95% CI) for MAD	Heterogeneity	
			I^2^	Q stat		I^2^	Q stat		I^2^	Q stat		I^2^	Q stat
Maternal Educational Status	Illiterate	0.4 (0.1, 1.8)	0.0%	0.11	1.2 (0.3, 4.7)	8.0%	3.26	1					
	Primary	1.6 (0.6, 4.2)	0.0%	1.28	1.4 (0.9, 2.2)	0.0%	3.05	1.6 (0.6, 4.2)	0.0%	1.28			
	Secondary	2.2 (0.6, 7.7)	0.0%	0.26	1.5 (0.8, 2.9)	0.0%	3.38	2.0 (0.8, 5.5)	0.0%	0.42			
	College^+^	1			1			1					
ANC follow-up	Yes	3.4 (1.5, 7.5) **	0.0%	1.19	1.8 (0.9, 3.5)	0.0%	2.67	3.4 (1.5, 7.5) **	0.0%	1.09			
	No	1			1			1					
PNC follow-up	Yes	1.9 (0.9, 3.8)	0.0%	2.77	2.3 (1.2, 4.3) **	0.0%	3.02	1.84 (0.9, 3.5)	0.0%	2.77	1.6 (0.5, 5.1)	0.0%	0.49
	No	1			1			1			1		
Counselling	Yes	2.6 (0.5, 12.9)	0.0%	0.15	2.3 (1.4, 3.9) *	0.0%	2.11	1.27 (0.3, 5.3)	0.0%	0.27			
	No	1			1			1					
Place of Delivery	HF	2.3 (1.0, 4.9) **	0.0%	2.56	2.1 (1.2, 3.7) *	67.5%	3.15	2.28 (1.0, 4.9) *	0.0%	3.06	2.2 (0.4, 12.1)	0.0%	0.45
	Home	1			1			1			1		
Mode of delivery	CS	1			1								
	Vaginal	3.3 (1.1, 10) *	0.0%	0.73	1.7 (1.2, 2.6) **	0.0%	1.00						
Residence	Rural	1.3 (0.5, 3.4)	0.0%	1.54	1.8 (1.1, 3.1) *	0.0%	7.86	1.27 (0.5, 3.4)	0.0%	1.54			
	Urban	1			1			1					
Child Age	0-1 month				4.4 (1.4, 13.6) *	0.0%	0.65						
	2-3 month				2.5 (1.2, 5.1) *	0.0%	4.11						
	4-6 month				1								
Maternal Age	15-24				1								
	25-34				1.9 (0.8, 4.8)	0.0%	1.71						
	>35				3.4 (1.3, 8.7)	0.0%	0.44						
Maternal Employment	Unemployed				1.8 (1.2, 2.7) **	92.6%	18.0	2.5 (0.7, 9.3)		0.26			
	Employed				1								
Sex of child	Male				1.3 (0.8, 1.9)	99.9%	6563						
	Female				1								
Husband support	Yes				2.9 (1.9, 4.4) ***	0.0%	0.69						
	No				1								
CF Demonstration	Yes										2.1 (1.3, 3.3) **	0.0%	0.06
	No										1		
Age of child in month	6-11										1		
	12-17										3.4 (0.7, 17.3)	0.0%	0.76
	18-23										2.7 (1.2, 6.1) *	0.0%	0.45
Wealth Index	Poor										1		
	Medium										1.6 (0.5, 4.7)	0.0%	0.75
	Rich										1.8 (0.7, 4.8)	0.0%	1.02

**N.B:** * indicates a significant level at p < 0.05, ** indicates a significant level at p < 0.01 *** indicates a significant level at p < 0.001.

Infants aged 0–1 and 2–3 months were over four times and three times more likely to be exclusively breastfed compared with infants aged 4–6 months (AOR = 4.4; 95% CI: 1.4, 13.6) and (AOR = 2.5; 95% CI: 1.2, 5.1), respectively. Mothers aged 35 years and above were about three times more likely to exclusively breastfeed their children compared with younger mothers (AOR = 3.4; 95% CI: 1.3, 8.7). Rural mothers had significantly higher odds of practicing exclusive breastfeeding compared with their urban counterparts (AOR = 1.8; 95% CI: 1.1, 3.1). Unemployed mothers were also 80% more likely to exclusively breastfeed than employed mothers (AOR = 1.8; 95% CI: 1.2, 2.7). Mothers who delivered in health facilities were twice as the likelihood of exclusively breastfeed compared with those who delivered at home (AOR = 2.1; 95% CI:1.2, 3.7).

Mothers who had normal (vaginal) deliveries were 70% more likely to exclusively breastfeed than those who delivered by cesarean section (AOR = 1.7; 95 CI:1.2, 1.2, 2.6). Postnatal care attendance was also positively associated with exclusive breastfeeding (AOR = 2.3; 95% CI: 1.2, 4.3). Additionally, mothers who received breastfeeding counseling were more likely to practice exclusive breastfeeding compared to those who didn’t (AOR = 2.3; 95% CI:1.4, 3.9) and mothers who received husband support were nearly three times more likely to exclusively breastfeed their infants (AOR = 2.9; 95% CI:1.9, 4.4) (Table 2).

Mothers who attended antenatal care were almost three times more likely to initiate complementary feeding at the recommended time compared with those who did not attend ANC (AOR = 3.4; 95% CI:1.5, 7.5). Likewise, mothers who delivered in health facilities had twice the likelihood of initiating complementary feeding on time compared with mothers who delivered at home (AOR = 2.3; 95% CI:1.1, 4.9) (Table 2).

Receiving nutrition education through practical demonstrations on complementary food preparation was also associated with better feeding practices; mothers exposed to such demonstrations were twice as likely to practice optimal feeding compared with their counterparts (AOR = 2.06; 95% CI:1.28, 3.31). Additionally, children aged 18–23 months were almost three times more likely to receive optimal feeding practices than those aged 6–11 months (AOR = 2.68; 95% CI:1.18, 6.13) (Table 2).

## Discussion

The aim of this study was to assess the pooled prevalence of feeding practices and the factors associated with them among children under two years of age in Ethiopia. The pooled prevalence of TIBF, EBF, TICF, and MAD were 64%, 59.56%, 59%, and 19.77%, respectively. A range of determinants were identified, including antenatal care, place and mode of delivery, postnatal care, maternal age and occupation, counseling, husband’s support, place of residence, maternal education, involvement in complementary food preparation demonstrations, and the child’s age.

The pooled prevalence of TIBF was 64%, which is in line with a SRMA done in Ethiopia (61.4%) [15], Sub-Saharan Africa (50.5%) [100] and Bangladesh [101]. According to WHO IYCF standards, it is considered good [102]. However, it was lower than the national and WHO IYCF recommendations [102,103]. But it was higher than the pooled prevalence reported among cesarean delivered mothers in Ethiopia (40.1%) and the recent estimate from 53 WHO European Region member countries (43%) [12,104]. These differences might be attributed to variations in study setting, methodological approaches, socio-demographic and economic characteristics, and health service utilization.

This study demonstrated that the pooled prevalence of EBF in Ethiopia was 59.56%. It is rated good according to the WHO IYCF standards (50%–89%). This result aligns with previous SRMA conducted in Ethiopia and Iran [14,15], the 2019 mini-EDHS report (59%), and a study in Southern Africa (56.57%) [15,105,106]. However, it is higher than the global EBF prevalence of exclusive breastfeeding (44%), SRMAs conducted in Ghana and Iran [107,108], and studies from Sub-Saharan Africa (36%), and Central Africa (53.48%) [105]. Conversely, the prevalence reported in this study is lower than findings from several regions of India and the Nepal DHS [109–111]. Such may be attributed to differences in socio-demographic and economic contexts, access to information, study periods, and methodological approaches.

This study also showed that more than half (59%) of infants received complementary feeding timely. According to WHO standards, it is rated as ‘fair’ (60–79%). This finding is consistent with a SRMA conducted in Ethiopia and a study conducted in South Asia [112,113], and it is congruent with the recent global estimate of 64.5% [114]. However, it is higher than a study conducted in five European Union countries (47%) [115]. These differences might be due to the differences in the study settings.

The pooled prevalence of minimum acceptable diet was 19.77%. This finding is consistent with a SRMA conducted in Ethiopia [18], as well as studies from South Asia [113] and Bangladesh [101], underscoring that many Ethiopian children continue to consume diets of inadequate quality [16]. However, the prevalence observed in this study is higher than the 2019 mini-EDHS report (11.3%), and the Ghana DHS [116], east Africa (11.58%) [117] and Sub-Saharan Africa (9.98%) [118]. In contrast, it was lower than reports from the Democratic Republic of Congo (33%) [119], and a study conducted in Indonesia (29%) [120]. These may reflect differences in the study methodologies, socio-demographic and economic contexts, access to diverse foods, and the time periods during which the studies were conducted.

Antenatal follow-up was identified as a predictor of TIBF. Mothers who attended ANC were three times more likely to initiate breastfeeding timely. This finding is consistent with evidence from a study done in Sub-Saharan African countries [121]. This might be because the nutrition education given during ANC visits which encourages skilled delivery and supports mothers in initiating breastfeeding soon after birth. However, this finding contrasts with studies done in Ethiopia and Namibia, where mothers who attended antenatal care were reported to be less likely to initiate breastfeeding on time [122,123]. This discrepancy may be due to variation in the interaction of health care providers during visits, as well as lack of coordinated responsibility, which could hinder the delivery of consistent messages to mothers.

This study identified place of delivery as a determinant of TIBF. Mothers delivered in a health facility were twice as likely to initiate breastfeeding on time compared with those who delivered at home. This finding is similar with evidence from a global systematic review and meta-analysis, as well as studies conducted in Iran, Namibia, and Nepal [108,123–125]. This might be because mothers who gave birth in a health facility were encouraged by health care providers through counseling and support on colostrum feeding, which enables them to initiate breastfeeding within the recommended time. Additionally, mothers delivering in health facilities are less likely to give prelacteal foods before initiating breastfeeding. On the other hand, studies from Ireland and the UK, and Canada revealed that home delivery was associated with higher odds of timely initiation of breastfeeding [126,127].

There was also a significant association between mode of delivery and TIBF. Mothers who delivered by cesarean section were 70% less likely to initiate breastfeeding timely as compared to vaginal delivery. This finding is consistent with studies conducted in Ghana and Kenya [128,129]. This may be due to post-operative care needs, pain, and fatigue, which can interfere with early skin-to-skin contact and immediate newborn care, both essential for timely initiation of breastfeeding. In contrast, a study from Central America reported a significant association between mode of delivery and timely initiation of breastfeeding [130]. This inconsistency may be due to the difference in socio-economic conditions and the quality and accessibility of health care services.

Younger children were more likely to be exclusively breastfed than older ones, with the likelihood of exclusive breastfeeding declining as age increases. This pattern is consistent with findings from several West African countries [131–133]. A possible explanation is that many mothers perceive breast milk alone as inadequate to meet the growing nutritional needs of older infants, prompting earlier introduction of complementary foods.

Maternal age was emerged as a significant predictor of exclusive breastfeeding. Mothers aged 35 years and older were three times more likely to exclusively breastfeed compared with those aged 15–24 years. This finding is in line with evidence from systematic reviews conducted in Brazil and Ghana [107,134]. One possible explanation is that younger mothers may perceive prolonged exclusive breastfeeding as affecting breast size or physical appearance, which could lead to earlier introduction of supplementary foods [135,136]. Furthermore, younger mothers may have limited awareness on the benefits of EBF, insufficient breastfeeding skills, or more frequent experiences of breastfeeding-related discomfort, all of which may contribute to lower exclusive breastfeeding practices. [137–139].

Maternal employment was a significant negative predictor of EBF. Employed mothers were 43% less likely to exclusively breastfeed their children compared with employed mothers. This finding is consistent with studies conducted across 19 developing countries [140], low and middle-income countries [141], and a SRMA from Ethiopia [14]. This could be because employed mothers return to work too early after birth due to short maternity leave, which can limit their ability to establish and sustain regular exclusive breastfeeding practices.

Conversely mothers from rural areas were 82% more likely to EBF their infants than urban residents. A similar result was reported by a previous SRMA conducted in Asia, Europe, and Africa [142]. This may be because rural mothers are predominantly housewives, are less likely to be engaged in formal employment, and tend to view breastfeeding as a natural maternal norm, unlike many of their urban counterpart. In addition, mothers living in rural areas are less exposed to and less familiar with breast milk substitutes promoted through media marketing compared with those in urban areas [143].

Mothers who delivered in a health facility were twice as likely to EBF their infants than home delivered mothers. This result was in line with studies done in Ethiopia, Tanzania and a systematic review and meta-analysis from Asia, Europe, and Africa [15,142,144]. This might be because women who delivered in a health facility could have a golden opportunity for nutrition education on the importance of EBF. In contrast, this finding contradicts with studies reported from Canada, Ireland and the UK, and Canada, which reported higher EBF rates among mothers who delivered at home [145]. This controversy could be explained by cultural differences among study participants and variations in awareness and understanding of exclusive breastfeeding practices [126,127].

Mothers who had normal delivery were twice as likely to EBF than those who delivered by cesarean section. This finding is consistent with a SRMA conducted in Iran [108]. This could be because of the nutrition counseling and breastfeeding support provided during labor and the immediate postpartum period, which encourage exclusive breastfeeding practices.

It was observed that mothers who received breastfeeding counseling were twice as likely to exclusively breastfeed their children compared with those who did not receive counseling, which was in accord with a SRMA performed by McFadden A et al. [146] and Ethiopia [147]. This may be due to the interactions with individual mothers during counselling, which enhance their knowledge, confidence, and decision-making regarding exclusive breastfeeding.

This study demonstrated that mothers who received support from their husband were almost three times more likely to exclusively breastfeed. This finding was corroborated by a SRMA conducted in Ghana and China [148,149]. This may be attributed to the emotional, physical, and practical support provided by husbands, which can strengthen maternal confidence, reduce stress, and ultimately enhance both the success and duration of exclusive breastfeeding. However, this finding contrasts with evidence from a systematic review and meta-analysis conducted by Sinha B. et a [150].

Mothers who attended postnatal care were more likely to EBF than their counterparts. This finding was in congruence with SRMA conducted in Ethiopia [151,152]. This may be because of the nutrition education and counseling received during the postnatal period, can help address breastfeeding difficulties, increase maternal confidence, and encourage social and family support, ultimately enabling mothers to maintain exclusive breastfeeding for six months.

Mothers who had antenatal care follow-up were more likely to initiate complementary feeding on time. This finding was consistent with a SRMA conducted in Ethiopia [112]. This could be because antenatal care visits provide an opportunity for mothers to receive information and counseling on appropriate complementary feeding practices from healthcare providers, thereby improving infant and young child feeding behaviors.

This study also showed that mothers who delivered at a health facility were more likely to initiate complementary feeding than home delivered mothers, which is in line with a SRMA in Ethiopia [153]. This could be attributed to the fact that mothers who gave birth in health institutions are more likely to receive counseling guidance from health professionals on appropriate child feeding practices.

It was also observed that children aged 18–23 months were more likely to receive a diet of good quality as per the recommendation compared to children aged 6–11 months. This finding was in line with studies done in Ethiopia, Ghana, and Uganda [154–157]. This may be because of the introduction of complementary feeding with a limited food item. Additionally, mothers may perceive that younger children have less developed digestive capacity for foods such as fruits, green leafy vegetables, and meat.

Mothers who received nutrition education through demonstration of complementary food preparation were more likely to achieve MAD as compared to their counterparts. This might be because such demonstration provides practical knowledge and skills on how to select, clean, and cook nutritious food preparation that meet the child’s nutritional needs. By observing and practicing food preparation during demonstrations, caregivers are more likely to adopt positive feeding behaviors, which enhances their confidence, promotes appropriate feeding practices, and supports the overall health and well-being of infants and young children.

This systematic review and meta-analysis have several limitations, including the exclusion of studies published in languages other than English. Furthermore, the high level of heterogeneity observed across the included studies may affect the reliability of the findings by introducing variability that can obscure true effects and potentially lead to misleading conclusions

## Conclusions

The pooled prevalence of child feeding practices in Ethiopia remains below both national and global infant and young child feeding recommendations. These suboptimal feeding practice act as an early warning signal, indicating heightened risks of malnutrition, morbidity, and mortality. Under such conditions, achieving the Sustainable Development Goals and the national aspiration of becoming a lower-middle-income country becomes increasingly challenging.

Key determinants influencing infant feeding practices include the child’s age, maternal age, maternal employment status, place of residence, antenatal care attendance, place of delivery, postnatal care utilization, and exposure to complementary food preparation demonstrations.

Strengthening infant feeding practices therefore requires a multifaceted approach. Priority actions should include enhancing nutrition education through practical demonstrations, extending maternity leave to six months or establishing workplace breastfeeding and feeding corners, promoting consistent attendance of antenatal and postnatal care services, increasing facility-based deliveries, and tailoring interventions to maternal age and residential context. Equal emphasis should be placed on promoting age-appropriate feeding practices to ensure optimal growth and development.

## Supporting information

S1 File Supp information. (RAR)

S1 Checklist PRISMA 2020 checklist. (DOCX)
