# Supplementary material for: Feeding practices of under-two children in Ethiopia: A systematic review and meta-analysis
Source: PLoS One. 2026 Feb 25;21(2):e0342932. doi: 10.1371/journal.pone.0342932 (PMC12935227; doi:10.1371/journal.pone.0342932)
Supplement: S1 Checklist — (DOCX) [file pone.0342932.s002.docx]

| **Section and Topic** | **Item #** | **Checklist item** | **Location where item is reported** |
| --- | --- | --- | --- |
| **TITLE** | | |  |
| Title | 1 | **Feeding Practices among Under-two Children in Ethiopia: A Systematic Review and Meta-Analysis** |  |
| **ABSTRACT** | | |  |
| Abstract | 2 | **Background:** globally, child feeding practice was suboptimal. This suboptimal feeding practice has an association with high burden of malnutrition. There is inadequate evidence on the aggregate infant feeding practices among under two children in Ethiopia. Thus, the purpose of this study was to assess the pooled prevalence and factors associated with child feeding practices.  **Method:** This systematic review and meta-analysis were done based on the PRISMA guidelines. Literature search was done in Scopus, HINARI, the Cochrane Library, and PubMed/MEDLINE. The heterogeneity of studies was tested by Cochran Q test and I^2^ test statistic. The data were analyzed using STATA/MP version 17.0. The prevalence was reported by the forest plot and a random-effects model of the DerSimonian-Laird method. Effect sizes (adjusted odds ratio) with a 95% CI were used to estimate the determinants of feeding practices.  **Results:** 107 articles were included in this study. The pooled prevalence of timely initiation of breastfeeding, exclusive breastfeeding, timely initiation of complementary feeding, and optimum feeding practice was 64% (95% CI: 50, 78), 58.57% (95% CI: 52.62, 64.53%), 60.51% (95% CI: 54.16, 66.87), and 20.36% (95% CI: 13.62, 27.04), respectively. Antenatal care (AOR=3.37%; 95% CI: 1.51, 7.52), giving birth at health facility (AOR = 2.28; 95% CI: 1.04, 4.98), normal delivery (AOR=0.34; 95% CI: 0.14, 0.87), and postnatal care were factors associated with TIBF. Age 0-1 (AOR=4.41; 95% CI: 1.43, 13.58) and 2-3 months (AOR=2.51; 95% CI: 1.23, 5.12), maternal age >35 (AOR=3.37; 95% CI: 1.31, 8.66), residence (AOR=1.82; 95% CI: 1.08, 3.08), maternal occupation (AOR=1.76; 95% CI: 1.16, 2.69), place of delivery (AOR=2.07; 95% CI:1.17, 3.68), CS delivery (AOR=1.74; 95 CI:1.18, 1.18, 2.57), postnatal care (AOR=2.29; 95% CI: 1.23, 4.25), counseling (AOR=2.32; 95% CI:1.36, 3.96) and husband support (AOR=2.91; 95% CI:1.93, 4.38) were factors associated with EBF. Antenatal care (AOR=3.38; 95% CI:1.51, 7.52) and place of delivery (AOR=2.28; 95% CI:1.04, 4.98) were the determinants of timely initiation of complementary feeding. Optimal infant feeding practice was associated with nutrition education through demonstrations (AOR=2.06; 95% CI:1.28, 3.31) and age of child 18-23 months (AOR = 2.68; 95% CI:1.18, 6.13)  **Conclusion:** This study revealed that infant feeding practices in Ethiopia were lower than the national and international infant feeding recommendations. Antenatal care, place of delivery, mode of delivery, postnatal care, maternal occupation, age of child, maternal age, place of residence, counseling, husband support, residence, education by demonstration of complementary food preparation and age of child were the determinants of infant feeding practices. Therefore, promoting infant feeding practice strategies should include nutrition education by demonstrations of complementary food, encouraging mothers to attend antenatal and postnatal care follow-up, increasing institutional delivery, increasing husband involvement in child feeding, considering the age of the mother and residence in child feeding, and promoting age specific child feeding practices. | 1-2 |
| **INTRODUCTION** | | |  |
| Rationale | 3 | Despite the benefits of optimal infant feeding practices, suboptimal feeding practices are still prevalent in many parts of the world, including Ethiopia. [According to the 2019 Ethiopian Demographic and Health Survey (EDHS), 59% of infants under six months of age were exclusively breastfed, and only 11.3% of children aged 6–23 months received a minimum acceptable diet](https://www.who.int/health-topics/infant-nutrition). This poor infant feeding practice is associated with a high prevalence of malnutrition. According to World Bank estimates, undernourished children have the possibility of losing over 10% of their lifetime earning capacity, which could have an impact on the country’s productivity  Although many studies were conducted on infant feeding practices, there was incongruities and inconsistencies across those studies. Previously conducted systematic reviews and meta-analyses on child feeding practices in Ethiopia had the following critique: A SRMA on timely initiation of breastfeeding contains an article extracted from the database, and conducted to see the effect of caesarean delivery on TIBF (14) and the effects of kangaroo mother care on TIBF among preterm and LBW infants  While a SRMA on exclusive breastfeeding was conducted by focusing on only one factor. A systematic review and meta-analysis of the minimum acceptable diet was done by including only nine studies and didn’t address the determinants of infant feeding practices  But the current study included over 107 articles and tried to assess the determinants of infant feeding practices, by considering four core IFCY indicators i.e., timely initiation of breastfeeding, exclusive breastfeeding, timely initiation of complementary feeding and minimum acceptable diet. This systematic review and meta-analysis will provide a comprehensive and rigorous synthesis of the available evidence on child feeding practices. The information generated could serve as an input for decision-making, resource allocation, and the design of new insight on the available interventions to improve IYCF practices as well as reduce the burden of poor child nutritional outcomes in Ethiopia. | 3-4 |
| Objectives | 4 | The aim of this study was to assess the pooled prevalence and factors associated with child feeding practices. | 4 |
| **METHODS** | | |  |
| Eligibility criteria | 5 | All studies that report prevalence and determinants of and factors associated with infant feeding practices in Ethiopia which are published in English were included. Cross-sectional and case-control studies were included in this study. Studies whose whole texts are unavailable after trying to contact the primary investigator within 3 months, descriptive studies, systematic reviews of the effects of an intervention, review articles, conference abstracts, and editorials were excluded from the study. | 6 |
| Information sources | 6 | This systematic review and meta-analysis was performed according to the Preferred Reporting Items for Systematic Review and Meta-Analysis (PRISMA) guidelines (19). All pertinent articles were comprehensively searched through MEDLINE (via PubMed), EMBASE, Cochrane Library, SCOPUS, HINARI, and Google Scholar. Gray literature was also included through manual search. The last date of search was conducted on September 2024. | 5 |
| Search strategy | 7 | **Searching terms**  Search concepts based around the below **CoCoPop** were developed for each database. Eligible studies were studies that included the following:  **Condition:** We included any study documenting infant feeding practice  **Context:** We included any study conducted in Ethiopia  **Population:** Studies targeting under two children feeding practice  prevalence OR magnitude AND (determinants) OR (“associated factors”) OR (“risk factors”) AND (“infant feeding practice”) AND (“exclusive breastfeeding”) OR (complementary feeding*) OR (“minimum acceptable diet”) OR (optimum nutrition) AND (Ethiopia). | 5 |
| Selection process | 8 | After obtaining the full text of all articles, duplicates were screened and removed from the citation manager (endnote). Data was extracted by three reviewers (SE, TB, & AT) by looking at titles, abstracts, and full articles. Article selection procedures were described by the PRISMA flow diagram (19). Studies that passed through this selection process were included in the study. Included and undecided studies were further assessed by reading their full text. Then, study characteristics (author, year of publication, region, target group, sample size, study design, response rate, and feeding practice), subject recruitment procedures, adjusted odds ratio and population characteristics were extracted by an extraction sheet developed with Microsoft Excel 2021. | 6 |
| Data collection process | 9 | Three reviewers were independently collected the published articles from MEDLINE (via PubMed), EMBASE, Cochrane Library, SCOPUS, HINARI, and Google Scholar | 5 |
| Data items | 10a | The outcome for this study were prevalence and factors associated with TIBF, EBF, TIBF and MAD. | 8-9 |
|  | 10b | study characteristics (author, year of publication, region, target group, sample size, study design, response rate, and feeding practice), subject recruitment procedures, adjusted odds ratio and population characteristics were extracted by an extraction sheet developed with Microsoft Excel 2021. Articles whose full article was not obtained after three months were excluded from the study. | 6 |
| Study risk of bias assessment | 11 | Risk of bias was assessed by funnel plots, Egger weighted regression, and Begg rank correlation tests | 7 |
| Effect measures | 12 | Prevalence of TIBF, EBF, TICF and MAD was reported by forest plot and odds ratio was used to report the effect measure(s). | 7 |
| Synthesis methods | 13a | After obtaining the full text of all articles, duplicates were screened and removed from the citation manager (endnote). Data were extracted by three reviewers (SE, TB, & AT) by looking at titles, abstracts, and full articles. Article selection procedures were described by the PRISMA flow diagram. Studies that passed through this selection process were included in the study. Included and undecided studies were further assessed by reading their full text. | 6 |
|  | 13b | Describe any methods required to prepare the data for presentation or synthesis, such as handling of missing summary statistics, or data conversions.  The prevalence of infant feeding practices was reported by the forest plot. To estimate the determinants of feeding practices among infants, effect size (adjusted odds ratio) with a 95% CI was pooled. The Cochrane Q statistic was used to test heterogeneity between study sites, and I^2^ statistic was used to quantify the magnitude of heterogeneity between studies. | 7 |
|  | 13c | Describe any methods used to tabulate or visually display results of individual studies and syntheses.  The prevalence of infant feeding practices was reported by the forest plot. To estimate the determinants of feeding practices among infants, effect size (adjusted odds ratio) with a 95% CI was pooled. The presence of heterogeneity was checked by the Galbraith plot and Forest plot. | 7 |
|  | 13d | Describe any methods used to synthesize results and provide a rationale for the choice(s). If meta-analysis was performed, describe the model(s), method(s) to identify the presence and extent of statistical heterogeneity, and software package(s) used.  Data were analyzed using STATA/MP version 17.0. The prevalence of infant feeding practices was reported by the forest plot. A DerSimonian and Laird random-effects model with a 95% confidence interval was used for high heterogeneity, and a fixed effects model with inverse variance methods was used for similar studies. The Cochrane Q statistic was used to test heterogeneity between study sites, and I^2^ statistic was used to quantify the magnitude of heterogeneity between studies. The Cochran Q statistic was considered significant if the P-value was < 0.05, and ***I^2^*** statistic, at least 50%, was considered suggestive of statistically significant heterogeneity. | 6-7 |
|  | 13e | The source of heterogeneity was tested by running meta-regression, subgroup analysis, and sensitivity analysis. Subgroup analysis was done by sex, age, and study setting (region). | 7 |
|  | 13f | Sensitivity analysis was done to assess robustness of the synthesized results. | 8 |
| Reporting bias assessment | 14 | Describe any methods used to assess risk of bias due to missing results in a synthesis (arising from reporting biases).  Publication bias was assessed by funnel plots, Egger weighted regression, and Begg rank correlation tests at p-values < 0.05 and trim and fill analysis was conducted for managing publication bias. | 7 |
| Certainty assessment | 15 | Describe any methods used to assess certainty (or confidence) in the body of evidence for an outcome. 95% CI was used to estimate the certainty. | 7 |
| **RESULTS** | | |  |
| Study selection | 16a | A total of 975 articles were retrieved by a literature search. After screening of title and abstract, 189 articles were identified as having information relevant to infant feeding practices. From the full article review, 107 articles were evaluated to have better quality data fitting the criteria for abstraction. Finally, 18 papers were included for timely initiation of breastfeeding, 38 papers were included to address exclusive breastfeeding, 25 papers were involved to assess timely initiations of complementary feeding, and 27 papers were included for optimum infant feeding practices. All included articles were full-text and done using cross-sectional and case control study designs. | 7-8 |
|  | 16b | 84 articles were excluded due to reason | N/A |
| Study characteristics | 17 | 107 studies were included in this study. | 8 |
| Risk of bias in studies | 18 | The presence of risk of bias was assessed using funnel plots, Egger weighted regression, and Begg rank correlation tests | 7 |
| Results of individual studies | 19 | The pooled prevalence of timely initiation of breastfeeding, exclusive breastfeeding, timely initiation of complementary feeding, and optimum feeding practice was 64% (95% CI: 50, 78), 58.57% (95% CI: 52.62, 64.53%), 60.51% (95% CI: 54.16, 66.87), and 20.36% (95% CI: 13.62, 27.04), respectively. | 8 |
| Results of syntheses | 20a | Publication bias was assessed subjectively using a funnel plot and objectively using Begg’s and Egger’s tests. The funnel plot (Figure 5) and Egg’s test (p = 0.011) of TIBF indicated the presence of publication bias, but Begg’s test showed no publication bias (p=0.289). So, trim and fill analysis was done for TIBF (Figure 8). The funnel plot of EBF (Figure 6) indicated the presence of publication bias, but Begg’s (p = 0.556) and Egger’s (p = 0.433) tests didn’t report publication bias. Based on the funnel plot (Figure 7), Begg’s (p = 0.056) and Egger’s (p = 0.06) tests of TICF, no publication bias was observed. The funnel plot (Figure 9) of optimal infant feeding practice showed the presence of publication bias but Begg’s (p = 0.89) and Egger’s (p = 0.934) tests revealed no publication bias. | 9 |
|  | 20b | Present results of all statistical syntheses conducted. If meta-analysis was done, present for each the summary estimate and its precision (e.g. confidence/credible interval) and measures of statistical heterogeneity. If comparing groups, describe the direction of the effect.  Antenatal care (AOR=3.37%; 95% CI: 1.51, 7.52), giving birth at health facility (AOR = 2.28; 95% CI: 1.04, 4.98), normal delivery (AOR=0.34; 95% CI: 0.14, 0.87), and postnatal care were factors associated with TIBF. Age 0-1 (AOR=4.41; 95% CI: 1.43, 13.58) and 2-3 months (AOR=2.51; 95% CI: 1.23, 5.12), maternal age >35 (AOR=3.37; 95% CI: 1.31, 8.66), residence (AOR=1.82; 95% CI: 1.08, 3.08), maternal occupation (AOR=1.76; 95% CI: 1.16, 2.69), place of delivery (AOR=2.07; 95% CI:1.17, 3.68), CS delivery (AOR=1.74; 95 CI:1.18, 1.18, 2.57), postnatal care (AOR=2.29; 95% CI: 1.23, 4.25), counseling (AOR=2.32; 95% CI:1.36, 3.96) and husband support (AOR=2.91; 95% CI:1.93, 4.38) were factors associated with EBF. Antenatal care (AOR=3.38; 95% CI:1.51, 7.52) and place of delivery (AOR=2.28; 95% CI:1.04, 4.98) were the determinants of timely initiation of complementary feeding. Optimal infant feeding practice was associated with nutrition education through demonstrations (AOR=2.06; 95% CI:1.28, 3.31) and age of child 18-23 months (AOR = 2.68; 95% CI:1.18, 6.13) | 1-2 |
|  | 20c | Present results of all investigations of possible causes of heterogeneity among study results.  A significant heterogeneity across studies was observed in timely initiation of breastfeeding (Chi^2^ = 1404.11; p < 0.0001; I^2^ = 99.7%),  exclusive breastfeeding (Chi^2^ = 3561.76; p < 0.0001; I^2^=98.9%, p=0.0001) and timely initiation of complementary feeding (chi-squared = 1664.94, I-squared= 98.6%%, p<0.001). The subgroup analysis was done by region and study setting, and still there is high heterogeneity. | 8 |
|  | 20d | The sensitivity analysis demonstrated that the quality score didn't affect the outcome of the meta-analysis and there was no significant difference in the overall pooled prevalence. | 8 |
| Reporting biases | 21 | Present assessments of risk of bias due to missing results (arising from reporting biases) for each synthesis assessed. N/A | N/A |
| Certainty of evidence | 22 | 95% confidence interval was used Certainty was assessed by | 7 |
| **DISCUSSION** | | |  |
| Discussion | 23a | Provide a general interpretation of the results in the context of other evidence.  The aim of this study was to assess the pooled prevalence and factors associated with feeding practices among under-two children in Ethiopia. Based on the findings of this study, the pooled prevalence of timely initiation of breastfeeding was 64%. This result was in line with a systematic review and meta-analysis done in Ethiopia (61.4%) (18), Sub-Saharan Africa (50.5%) (72) and Bangladesh (73). TIBF in Ethiopia was rated good as per the WHO IYCF standards (74). However, it was lower than the national and WHO IYCF recommendations (74, 75). But it was higher than a systematic review and meta-analysis done among cesarean delivered mothers in Ethiopia (40.1%) and a recent estimate of 53 WHO European Region member countries (43%) (15, 76). This difference might be because of the study setting, methodological differences, variation in socio-demographic and economic characteristics, and health service utilization.  This study demonstrated that the pooled prevalence of exclusive breastfeeding in Ethiopia was 58.57%. EBF is also rated good as compared to the WHO IYCF standards (50%–89%). This result was consistent with a systematic review and meta-analysis conducted in Ethiopia and Iran (17, 18), the 2019 mini-EDHS report (59%), and a study done in Southern Africa (56.57%) (18, 77, 78). But it was higher than the global prevalence of EBF (44%), a systematic review and meta-analysis conducted in Ghana and Iran (79, 80), studies conducted in Sub-Saharan African countries (36%), and Central Africa (53.48%) (77). On the other hand, it was lower than studies conducted in Indian regions and the Nepal Demographic and Health Survey (81-83). This variation could be because of the socio-demographic and economic differences, access to information, the study period, and methodological differences.  This study also showed that more than half (59.15%) of infants in Ethiopia were initiated complementary feeding on time. Based on the World Health Organization (WHO) standard, the national prevalence of TICF is rated as ‘fair’ (60 to 79%). This finding was consistent with a systematic review and meta-analysis conducted in Ethiopia and a study conducted in South Asia (84, 85). It also congruences with the recent global estimate (64.5%) (86). While it was higher than a study conducted in five European Union countries (47%) (87). This difference might be due to the differences in the study setting and design.  The pooled prevalence of optimal infant feeding practice was 20.36%. This result is consistent with a systematic review and meta-analysis conducted in Ethiopia (19), studies conducted in America, Asia, and Africa (21%) (88), South Asia (85), and Bangladesh (73). This implies that most Ethiopian children’s diet was of low quality (89). But it was higher than the 2019 mini-EDHS report (11.3%), and Ghanian DHS (90), east Africa (11.58%) (91) and Sub-Saharan Africa (9.98%) (92). Whereas, it was lower than studies conducted in Democratic Republic of Congo DHS report (33%) (93), and Indonesia (29%) (94). This variation could be because of the differences in methodology, socio-demographic and economic characteristics, and the period gap in which studies were conducted.  Antenatal follow up was found to be a predictor of timely initiation of breastfeeding. Mothers who had antenatal care follow up were three times more likely to initiate breastfeeding on time as compared to their counterparts. This finding was similar to a study done in Sub-Saharan African countries (95). This might be because the nutrition education given during ANC visits will promote skilled delivery and help mothers initiate breastfeeding soon after birth. However, this finding contradicts with a study done in Ethiopia and Namibia, which stated that mothers who had antenatal follow up were less likely to initiate breastfeeding on time (96, 97). This variation might be due to the interaction of different health care providers at different visits, and lack of shared responsibility may hinder the transfer of consistent messages to mothers.  This study revealed that place of delivery was a determinant factor for timely initiation of breastfeeding. Mothers who gave birth at health facility were twice as likely to initiate breastfeeding as those who delivered at home. This finding is consistent with a systematic review and meta-analysis of world literature and Iran, and studies done in Namibia and Nepal (80, 97-99). This might be because mothers who gave birth at a health facility were encouraged by health care providers through counseling and support on colostrum feeding, which enables them to initiate breastfeeding within the recommended time. The other possible explanation could be that mothers who gave birth at a health facility had less chance of giving Prelacteal food to the newborn prior to initiation of breastfeeding. On the other hand, studies from Ireland and the UK, and Canada revealed that home delivery meaningfully amplified the odds of TIBF (100, 101).  There was a significant association between mode of delivery and timely initiation of breastfeeding. Mothers who delivered through a cesarean section were 66% less likely to timely initiate breastfeeding as compared to vaginal delivery. This result was consistent with studies conducted in Ghana and Kenya (102, 103). This may be because of the post-operative care, pain, and fatigue that possibly disrupt early skin-to-skin contact and the immediate new-born care that supports TIBF. Another study in Central America showed that mode of delivery does not affect TIBF (104). This inconsistency may be due to the difference in socio-economic status and health care services.  Younger children were more likely to be exclusively breastfed than older children. As the age of the child increases, the probability of exclusive breastfeeding decreases. This finding was consistent with studies conducted in Western African countries (105-107). This could be because, as the child’s age increases, their mother perceives that breast milk alone can’t meet their nutritional requirements. As the child’s age increases, mothers are more likely to introduce complementary foods.  Maternal age is a significant predictor of exclusive breastfeeding among Ethiopian children. Mothers aged 35 years and older were three times more likely to exclusively breastfeed their children as compared to those aged 15-24 years. This result was consistent with a systematic review conducted in Brazil and Ghana (79, 108). This may be due to the perception of young mothers about the effect of longer exclusive breastfeeding time on the size of the breast and their beauty, hence, they usually start supplementary feeding early (109, 110). It might also be due to lack of awareness about the benefits of exclusive breastfeeding, inadequate breastfeeding skills, or painful breastfeeding experiences (111-113).  Maternal employment was a significant predictor of exclusive breastfeeding. This study revealed that employed mothers were 43% less likely to exclusively breastfeed their children than employed mothers. This finding is consistent with studies conducted in 19 developing countries (114) and low and middle-income countries (115), and a systematic review and meta-analysis conducted in Ethiopia (17). This could be because employed mothers return to work too early after birth due to short maternity leave, which hinders mothers’ ability to establish and maintain a consistent breastfeeding routine.  Mothers from rural areas were 82% more likely to exclusively breastfeed their infants than urban residents. A similar result was reported by a previous systematic review and meta-analysis conducted in Asia, Europe, and Africa (116). This might be because of the increased access to health facilities and nutrition education on exclusive breastfeeding. In addition, mothers living in rural areas have less effect and acquaintance with breastmilk substitutes circulated through media marketing compared to urban areas (117).  Mothers who delivered at a health facility were two times more likely to exclusively breastfeed than home births. This result was in line with studies done in Ethiopia, Tanzania and a systematic review and meta-analysis conducted in Asia, Europe, and Africa (18, 116, 118). This might be because women who deliver at health facilities could have a golden opportunity for nutrition education on the importance of EBF. On the other hand, our finding contradicts with a study reported from Canada, which stated that home delivered mothers were more likely to exclusively breastfeed their children as compared to institutional delivery (119). This controversy could be explained by the study participants’ cultural variation and level of understanding about the importance of exclusive breastfeeding. On the other hand, recent studies in Ireland and the UK, and Canada reveal that home delivery meaningfully raises the probabilities of EBF (100, 101).  Mothers who have normal delivery were two times more likely to exclusively breastfeed than cesarean section delivered mothers. This finding was consistent with a systematic review and meta-analysis conducted in Iran (80). This could be because of the nutrition education given during labor, which encourages mothers to exclusively breastfeed their children.  Mothers who got breastfeeding counselling were two times more likely to breastfeed exclusively their children than their counterparts. This finding is in line with a systematic review and meta-analysis performed by McFadden A et al. (120) and Ethiopia (121). This may be due to the interactions with individual mothers during counselling, which will help boost their decision-making about exclusive breastfeeding.  Mothers who got husband support were almost three times more likely to exclusively breastfeed than their counterparts. This finding was corroborated by a systematic review and meta-analysis conducted in Ghana and China (122, 123). This might be because the intimate contact of the husband gives more physical and emotional support for the mother, which is more likely to improve the success and duration of exclusive breastfeeding. But this finding was contradicted by a systematic review and meta-analysis conducted by Sinha B, et al. (124).  Mothers who attended postnatal care were almost three times more likely to exclusively breastfeed than their counterparts. This finding was in congruence with a systematic review and meta-analysis done in Ethiopia (125, 126). This may be because of the nutrition education that the mother received during the postnatal period which may comfort breastfeeding trouble, increase maternal confidence, and encourage social/family support, which led the mother to continue EBF for 6 months.  Mothers who had antenatal care follow up were almost three times more likely to initiate complementary feeding on time than their counterparts. This finding is consistent with a systematic review and meta-analysis conducted in Ethiopia (84). This could be due to visiting a healthcare facility during pregnancy provides an opportunity to obtain information and counseling on complementary feeding by healthcare providers, which improves infant and young children’s feeding practices.  Mothers who delivered at a health facility were two times more likely to initiate complementary feeding than home delivered mothers. This is in line with a systematic review and meta-analysis conducted in Ethiopia (127). This could be attributed to the fact that mothers who gave birth in health institutions would receive adequate information from health professionals about child feeding practices.  Age of the child was found to be positively associated with optimum nutrition. The findings of this study showed that children in the age range of 18–23 months were more likely to receive a diet of good quality as per the recommendation compared to children aged 6-11 months. This finding was in line with studies done in Ethiopia, Ghana, and Uganda (128-131). This may be because of the start of complementary feeding with only limited items. Mothers might also be able to perceive that the younger the children, the weaker their intestinal capacity to digest fruits, green leafy vegetables, and meat.  Mother who gets nutrition education through demonstration of complementary food preparation were two times more likely to get optimum nutrition as compared to their counterparts. This might be because a complementary food preparation demonstration provides practical knowledge and skills on how to select, clean, and cook nutritious food preparation to meet the child’s nutritional needs. Observing and practicing food preparation during demonstrations can lead to positive behavior change, which empowers caregivers, promotes healthy feeding practices, and contributes to the overall well-being of infants and young children. | 9-15 |
|  | 23b | Discuss any limitations of the evidence included in the review.  The limitation of this study was exclusion of articles published other than English language. | 16 |
|  | 23c | Discuss any limitations of the review processes used.  N/A |  |
|  | 23d | Discuss implications of the results for practice, policy, and future research.  The pooled prevalence of child feeding practice in Ethiopia was lower than the national and global infant and young child feeding recommendations. This low feeding practice is a yellow light signaling a higher risk of malnutrition, morbidity, and mortality. With this rate of feeding practice, it is difficult to achieve the sustainable development goal and the vision of being a lower- and middle-income country by 2025. | 16 |
| **OTHER INFORMATION** | | |  |
| Registration and protocol | 24a | Provide registration information for the review, including register name and registration number, or state that the review was not registered.  This systematic review and meta-analysis were registered in PROSPERO with a CRD number of 42023489496. | 6 |
|  | 24b | Indicate where the review protocol can be accessed, or state that a protocol was not prepared.  The protocol was not prepared | 6 |
|  | 24c | Describe and explain any amendments to information provided at registration or in the protocol.  For the registration purpose, the protocol was prepared and submitted to Prospero. | 6 |
| Support | 25 | Describe sources of financial or non-financial support for the review, and the role of the funders or sponsors in the review.  Nu funding was obtained for this study. | 17 |
| Competing interests | 26 | Declare any competing interests of review authors.  Authors declare that there is no conflict of interest. | 17 |
| Availability of data, code and other materials | 27 | Report which of the following are publicly available and where they can be found: template data collection forms; data extracted from included studies; data used for all analyses; analytic code; any other materials used in the review.  All the required data has been included in the manuscript. | 16 |

*From:*  Page MJ, McKenzie JE, Bossuyt PM, Boutron I, Hoffmann TC, Mulrow CD, et al. The PRISMA 2020 statement: an updated guideline for reporting systematic reviews. BMJ 2021;372:n71. doi: 10.1136/bmj.n71. This work is licensed under CC BY 4.0. To view a copy of this license, visit <https://creativecommons.org/licenses/by/4.0/>
